# Tuning the *in vitro* sensing and signaling properties of cyanobacterial PII protein by mutation of key residues

**DOI:** 10.1038/s41598-019-55495-y

**Published:** 2019-12-12

**Authors:** Khaled A. Selim, Michael Haffner, Björn Watzer, Karl Forchhammer

**Affiliations:** Interfaculty Institute of Microbiology and Infection Medicine, Department of Organismic Interactions, Eberhard Karls University of Tübingen, Auf der Morgenstelle 28, 72076 Tübingen, Germany

**Keywords:** Biochemistry, Microbiology, Molecular biology

## Abstract

PII proteins comprise an ancient superfamily of signal transduction proteins, widely distributed among all domains of life. In general, PII proteins measure and integrate the current carbon/nitrogen/energy status of the cell through interdependent binding of ATP, ADP and 2-oxogluterate. In response to effector molecule binding, PII proteins interact with various PII-receptors to tune central carbon- and nitrogen metabolism. In cyanobacteria, PII regulates, among others, the key enzyme for nitrogen-storage, N-acetyl-glutamate kinase (NAGK), and the co-activator of the global nitrogen-trascription factor NtcA, the PII-interacting protein-X (PipX). One of the remarkable PII variants from *Synechococcus elongatus* PCC 7942 that yielded mechanistic insights in PII-NAGK interaction, is the NAGK-superactivating variant I86N. Here we studied its interaction with PipX. Another critical residue is Lys58, forming a salt-bridge with 2-oxoglutarate in a PII-ATP-2-oxoglutarate complex. Here, we show that Lys58 of PII protein is a key residue for mediating PII interactions. The K58N mutation not only causes the loss of 2-oxogluterate binding but also strongly impairs binding of ADP, NAGK and PipX. Remarkably, the exchange of the nearby Leu56 to Lys in the K58N variant partially compensates for the loss of K58. This study demonstrates the potential of creating custom tailored PII variants to modulate metabolism.

## Introduction

The PII signal-transduction superfamily is widespread in all domains of the life, representing one of the largest and most ancient families of signaling proteins in nature^1,2^. Signaling proteins of the PII superfamily are characterized by their highly conserved trimeric structure, consisting of a triangular core of β-sheets from the ferredoxin-like fold of the three subunits^1–3^. Canonical PII proteins are involved in the regulation of various carbon and nitrogen metabolism related processes^1,2,4^. For this purpose, PII acts as an energy/carbon/nitrogen sensor: binding of metabolites as well as post-translational modifications enable PII to integrate different signals^1,2,4–8^. Based on this input signals, PII triggers a specific output signal by interacting with target proteins including regulatory proteins, transcription factors, enzymes of carbon/nitrogen metabolism or transporters^1,2,4^. The competitive binding of ATP or ADP and synergistic binding of 2-oxogluterate (2-OG) allows PII to estimate the current energy and nitrogen/carbon status of the cell^4–9^. Canonical PII proteins are highly conserved homo-trimeric proteins with three characteristic loop regions (T-, B- and C-loops)^1,2,4^. These loop regions are located near the inter-subunit clefts and play a major role in intramolecular signaling, protein-protein interactions and ligand binding^1,2,4–7^.

In cyanobacteria and plants, the major interaction partners of PII described so far are the controlling enzyme or arginine synthesis, N-acetyl-L-glutamate kinase (NAGK)^6,10–13^, the BCCP-subunit of Acetyl-CoA Carboxylase (ACCase)^14^. Restricted to cyanobacteria only, the transcriptional co-activator PipX^9,15–18^ as well as the major nitrogen-transport systems^19^ for uptake of nitrate (NRT), ammonium (AMT) and urea (URT). PipX acts as a transcriptional co-activator of the master transcription factor of nitrogen-regulated genes, NtcA^8,15–18^. Under sufficient nitrogen supply, indicated by low 2-OG levels, PII forms an ATP-dependent activating complex with NAGK and in addition, forms a complex, preferentially in the ADP-bound state, with PipX, thereby competing with NtcA-PipX complex formation^8^. Formation of the PII-NAGK complex increases the catalytic efficiency of NAGK and decrease the feedback inhibitory effect of arginine to NAGK^10,11^. Under conditions of high 2-OG levels (poor nitrogen supply), the ATP-dependent binding of 2-OG to PII causes strong conformational changes in T-loop^5^, which in turn impairs the interaction of PII proteins with both NAGK^6,10^ and PipX^8,15,16^. Binding of PII in the ATP-bound state to the BCCP-subunit of ACCase inhibits the ACCase activity^14,20,21^. In all these cases, the PII proteins exert their regulatory function through binding the small effector molecules ATP/ADP and 2-OG, which induces conformational changes on the flexible surface exposed T-loop, the PII’s major protein-interaction structure^1,2,7^.

Previously, we showed that replacement of B-loop residue Ile86 with Asn causes a conformational change caused by additional hydrogen bonding, that leads to folding of the PII T-loop in a bent conformation similar to the T-loop conformation in the PII-NAGK complex^6^. As a consequence of this mutation, the PII (I86N) variant has low affinity towards 2-OG and binds constitutively to NAGK *in vitro* and *in vivo*, leading to an accumulation of arginine and cyanophycin^6,22^. From various PII crystal structures, Lys58 is known to reside in various alternative states: in the ATP and ADP complexes, it interact with the B-loop through direct interaction with Gly87^5,6,23^, and in the PII-NAGK complex, it interacts with the T-loop via salt bridge with Glu44^6,12^. By contrast, in the PII-Mg^2+^-ATP-2-OG complex, Lys58 forms a salt-bridge to the gamma-carboxyl group of 2-OG^5,24^. To gain deeper insights in the signaling mechanism of PII from cyanobacterium *Synechococcus elongatus* PCC 7942 (hereafter as *Se*PII), we investigated the influence of PII (I86N) mutation on the interaction with PipX and furthermore, checked the importance of Lys58 for PII functions, in particular, for the PII-interaction with NAGK and PipX.

## Results

### Lys58 is crucial for ligand binding

Formation of the PII-NAGK complex requires a compact bending of the protruding PII T- loop. The T-loop is inserted in the interdomain cleft of the NAGK subunits for anchoring PII on NAGK^11^. In this tightly folded conformation, the T-loop residue Glu44 forms a salt-bridge with the highly conserved residue Lys58^6,12^. The same Lys58 residue plays a key role for 2-OG binding to the PII-Mg^2+^-ATP complex, through formation of a salt-bridge with the C5-carboxyl-group of 2-OG^5,24^. When the 2-OG levels increase in the cell, the PII-NAGK complex must dissociate to allow PII-2-OG interaction^1^. Therefore, the intramolecular Lys58-Glu44 salt bridge must break and instead, the salt bridge between Lys58 and 2-OG forms, arresting the T-loop in a novel conformation that precludes NAGK interaction^5,12^ (summarized in^1,25^). To directly assess the importance of PII Lys58 for NAGK complex formation, we created a *Se*PII (K58N) variant, where Lys58 was replaced by Asn, and therefore the Lys58-Glu44 salt bridge cannot form. We decided to mutate the conserved Lys58 residue to Asn because this replacement is found in the PII-like protein SbtB^3^ (PDB: 5O3R), as revealed by structure-based sequence alignment between *Se*PII (PDB: 2XUL) and *Synechocystis* SbtB^3^ (Supplementary Fig. S1).

**Figure 1:**
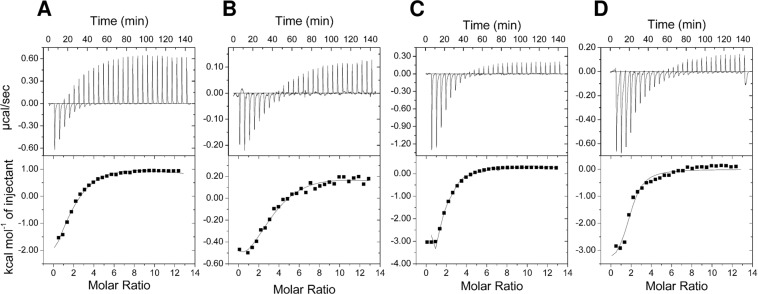
ITC analysis of small effector molecule binding to PII (K58N) and (K58N/L56K) variants. The upper panels show the raw isothermal data in the form of the heat effect during the titration of PIIs solution with ligands. The lower panels show the binding isotherm and the best-fit curve according to the three sequential binding sites model. Titration of 33 µM (trimer concentration) of PII variants: (**A**) (K58N) with 2 mM ATP, (**B**) (K58N) with 2 mM ADP, (**C**) (K58N/L56K) with 2 mM ATP, and (**D**) (K58N/L56K) with 2 mM ADP.

The binding properties of *Se*PII (K58N) variant in comparison to the wild-type *Se*PII (WT) protein were analyzed by isothermal titration calorimetry (ITC) (Fig. 1). As expected, the PII variant K58N lost the ability to bind 2-OG (Supplementary Fig. S1), whereas ATP was still able to bind (Fig. 1A), albeit weaker than the *Se*PII (WT) protein (Table 1). The binding of ADP was even more strongly impaired (Fig. 1B), yielding only very weak isotherm signals. This agrees with the fact that the Lys58 residue is crucial for establishing a stable T-loop conformation of the ADP-complex through hydrogen-bonding interaction with the Gln39 side chain^7,23^. In the PII-ADP structures (PDBs: 4CNZ and 4C3K), the side chain of residue Leu56 also approaches the Gln39 side chain. We, therefore, asked the question whether replacement of Leu56 by Lys might be able to compensate the K58N mutation. To test this hypothesis, we created a double point mutation variant *Se*PII (K58N/L56K). The *Se*PII (K58N/L56K) variant was first tested for its effector molecule binding properties using ITC. This PII variant was still unable to bind 2-OG (Supplementary Fig. S1), but the binding affinity toward ATP was enhanced in comparison to the *Se*PII (K58N) variant (Fig. 1, compare C with A). Especially, the first biding site was occupied with very high affinity. The ADP binding events to the *Se*PII (K58N/L56K) variant induced a strong isotherm, and in particular, the affinity for the second binding site was strongly enhanced (Table 1, Fig. 1, compare D with B). As can be deduced from the binding isotherms, the binding enthalpy for ATP and ADP binding to *Se*PII (K58N/L56K) variant was stronger than to *Se*PII (K58N) variant (Fig. 1, compare A with C and B with D). These results indicate that a re-location of the Lys-residue by two amino acid positions does not rescue 2-OG binding but positively influences adenyl nucleotide binding, in agreement with the fact that Lys58 is a direct ligand to 2-OG^5,24^ but is only indirectly involved in adenyl-nucleotide binding^7,23^ through affecting the T-loop conformation.

**Table 1:** Dissociation constants (*K*_*d*_) for binding ATP, ADP and 2-OG to the recombinant *Se*PII variants.

Titrant/Protein	Three-sites binding model		
	*K*_*d1*_ (µM)	*K*_*d2*_ (µM)	*K*_*d3*_ (µM)
**ATP**			
PII (WT)^9^	(7.5)	(15.9)	(85.4)
PII (K58N)	27.3	90.2	1445.1
PII (K58N/L56K)	0.5	43.3	1016.26
**ADP**			
PII (WT)^9^	(10.6)	(19.3)	(133.4)
PII (K58N)	86.2	109.2	680.3
PII (K58N/L56K)	96.2	42.9	719.4
**2-OG in presence of ATP**			
PII (WT)^9^	(2.9)	(6.4)	(83.0)
PII (K58N)	NB		
PII (K58N/L56K)	NB		

The raw isothermal data were fitted according to three sequential binding sites model for PII trimer. NB: No binding.

### PII variants K58N and K58N/L56K are still able to activate NAGK

**Figure 2:**
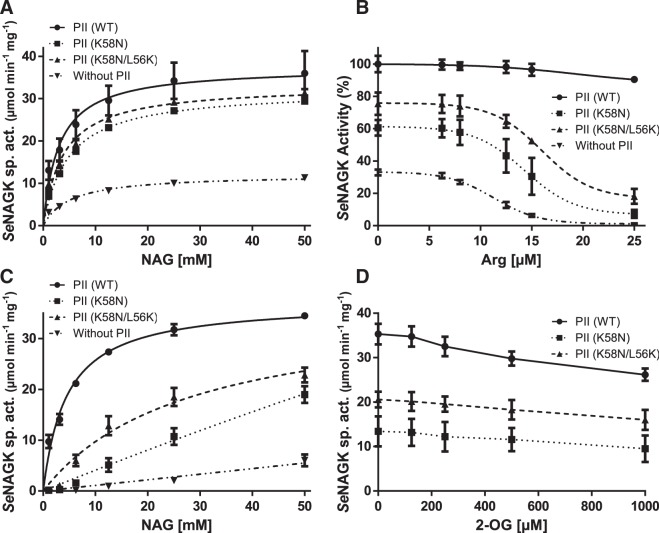
Response of *Se*NAGK activity towards *Se*PII (WT) or variants (K58N) and (K56N/L56K). (**A**) *Se*NAGK activity with or without different PII variants, as indicated. NAG was used as a variable substrate. (**B**) Arginine-feedback inhibition of *Se*NAGK activity in the presence or absence of different variants of PII protein, as indicated. Data were fitted according to a sigmoidal dose-response curve using GraphPad Prism to estimate the IC_50_ for arginine. (**C**) *Se*NAGK activity with or without different PII variants in presence of 11 µM arginine, as indicated. NAG was used as a variable substrate. (**D**) Effect of 2-OG on PII-promoted activation of *Se*NAGK in presence of 11 µM arginine, as indicated. SD as indicated by error bars, represents triplicate independent measurements.

Next, we wanted to investigate the ability of *Se*PII variants (K58N and K58N/L56K) to interact and activate NAGK. The PII-based activation of NAGK was tested using a coupled enzyme assay^10^ with recombinant NAGK proteins deriving from strains *S. elongatus* PCC7942 (*Se*NAGK) and *Synechocystis* sp. PCC 6803 (*Sc*NAGK). The catalytic activity of the *Se*NAGK was determined with NAG (N-acetyl-glutamate) as a variable substrate in presence or absence of different PII protein variants (kinetic constants are listed in Table 2). Both PII variants were able to activate *Se*NAGK (Fig. 2A), but weaker than *Se*PII (WT). With *Sc*NAGK, the *Se*PII (K58N) variant was not able to activate *Sc*NAGK (Supplementary Fig. S2) whereas the *Se*PII (K58N/L56K) variant was again able to partially activate *Sc*NAGK (Supplementary Fig. S2). This shows that interaction of *S. elongatus* PII with the non-cognate *Sc*NAGK (from *Synechocystis*) is in principle less robust against variations in the amino acid sequence. However, re-location of Lys58 to position 56 helps PII to adopt a compensatory conformation, which allows productive interaction with the non-cognate NAGK partner.

**Table 2:** Kinetics of *Se*NAGK in presence or absence of PII variants.

	*K*_*m*_ (mM) for NAG	*V*_*max*_
**In absence of feedback inhibitor Arg**		
*Se*NAGK	5.1 ± 0.9	12.1 ± 0.7
*Se*NAGK + *Se*PII (WT)	2.9 ± 0.6	37.5 ± 1.6
*Se*NAGK + *Se*PII (K58N)	4.8 ± 0.5	32.1 ± 0.9
*Se*NAGK + *Se*PII (K58N/L56K)	3.8 ± 0.6	33.2 ± 1.5
**In presence of feedback inhibitor Arg** (**11** **µM**)		
*Se*NAGK	NF	
*Se*NAGK + *Se*PII (WT)	4.5 ± 0.7	37.3 ± 1.5
*Se*NAGK + *Se*PII (K58N)	NF	
*Se*NAGK + *Se*PII (K58N/L56K)	27.8 ± 10.5	36.7 ± 6.9

The kinetics of *Se*NAGK were determined in absence or presence of arginine IC_50_ (11 µM). NF: not fitted to Michaelis-Menten kinetics.

### Tight complex formation of PII-NAGK is required to relieve arginine feedback inhibition

The relief of NAGK from arginine feedback inhibition through PII-NAGK complex formation is the rate-limiting step for the metabolic switch of the arginine biosynthetic pathway^9,10,13^. To examine whether the different PII variants would relief NAGK from arginine inhibition, we assessed *Se*NAGK activity at fixed concentration of NAG (40 mM) in presence of different concentrations of arginine with and without different PII protein variants. Arginine feedback inhibition of non-complexed *Se*NAGK occurred with a half maximal inhibitory concentration (IC_50_) of 11 µM (Fig. 2B). As expected, the arginine inhibitory effect on *Se*NAGK was strongly released in presence of *Se*PII (WT) protein (Fig. 2B). Intriguingly, both *Se*PII variants (K58N and K58N/L56K), which were able to activate *Se*NAGK, failed to markedly relieve *Se*NAGK from arginine feedback inhibition, although a subtle effect was observed (with IC_50_ values of 14.2 or 15.7, as compared to 11 µM for *Se*NAGK alone) (Fig. 2B). Together, it appeared that the double point mutation variant activated *Se*NAGK more strongly and was slightly more efficient in relieving *Se*NAGK from arginine inhibition than the single point mutation (K58N) (Fig. 2B).

The catalytic activity of the *Se*NAGK with NAG as a variable substrate in the presence of 11 µM arginine (which corresponds to the IC_50_ of *Se*NAGK alone) with or without different PII variants was determined to discern the two PII mutant variants (kinetic constants are listed in Table 2). In this assay, the *Se*PII (K58N/L56K) variant was much more efficient than the *Se*PII (K58N) variant to activate *Se*NAGK (Fig. 2C). Due to the low activity, the kinetic constants for the activation of *Se*NAGK by *Se*PII (K58N) variant could not be calculated, as the data could not be fitted to Michaelis-Menten equation. Similar results were obtained using the *Sc*NAGK enzyme (Supplementary Fig. S2). Together these results reinforce that mutation of Leu56 to Lys56 in the *Se*PII (K58N/L56K) variant partially compensates the function of Lys58 in stabilizing PII-NAGK complex, most likely by approaching again the Glu44 residue of the T-loop (PDBs: 2XBP and 2V5H).

Next, we determined the response of the various PII-NAGK complexes to 2-OG in presence of 11 µM arginine, the IC_50_ of free *Se*NAGK. As shown in Fig. (2D), the addition of 2-OG had a negligible influence on both variants of PII proteins confirming the inability of Lys58 mutant to bind 2-OG effectively (Fig. 2D). Since in presence of 11 µM arginine the activity of NAGK is only weakly inhibited in presence of *Se*PII (WT), the effect of 2-OG is modest. However, in presence of 25 µM arginine, the complex of *Se*NAGK-*Se*PII (WT) showed the typical inhibitory effect upon 2-OG addition^6,10^ (Supplementary Fig. S2).

**Figure 3:**
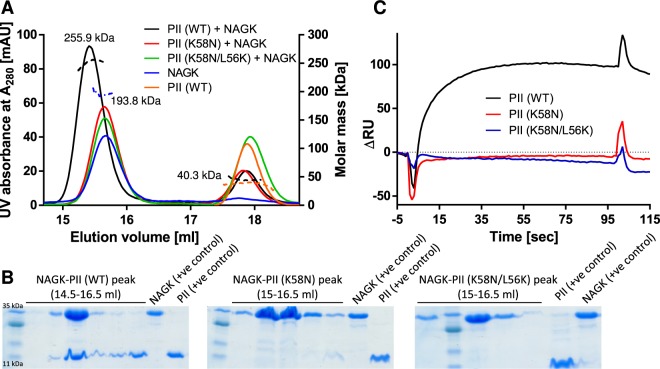
NAGK-PII complex formation between *Se*NAGK and *Se*PII variants using SEC-MALS and SPR. (**A**) SEC-MALS analysis indicates dissociation of NAGK-PII (K58N) and NAGK-PII (K58N/L56K) complexes through gel filtration run, while NAGK-PII (WT) forms a strong complex with higher molar mass. The eluted peaks from SEC runs were collected and subjected to SDS-PAGE. (**B**) The eluted protein fractions between 14.5–16.5 ml (as indicated) corresponding to the SEC runs shown in (A) were collected and subjected to SDS-PAGE followed by Coomassie blue staining. The SDS-PAGE analysis confirmed the presence of both PII (WT) and NAGK proteins in the NAGK-PII (WT) complex peak, while NAGK was present alone in both of NAGK-PII (K58N) and NAGK-PII (K56N/L56K) runs, indicating that for both PII variants the complex dissociated during SEC. (**C**) SPR analysis of NAGK-PII complex formation. His_6_-NAGK was immobilized to the Ni-NTA sensor chip and different PIIs variants (as indicated) were injected to determine changes in resonance units (RUs) due to NAGK-PII complex formation. PII (WT) efficiently bound to NAGK while both of PII variants (K58N and K58N/L56K) did not show any response.

Form the previous enzyme assays, it turned out that both PII variants were still able to interact with NAGK, but this interaction seemed to be impaired to different extent, with the (K58N/L56K) variant partially compensating the impairment of the K58N variant. To further confirm this assumption, we first tried to isolate PII-NAGK complexes of different variants of PII (K58N) and (K58N/L56K) using analytical gel-filtration coupled with multi angle light scattering (MALS) (Fig. 3A). *Se*PII (WT) protein forms a stable complex with *Se*NAGK with a molar-mass of 275.4 kDa, corresponding to two-PII trimers (each trimer of ≈40.8 kDa) sandwiching one hexameric *Se*NAGK (≈194 kDa)^12^. In this experimental setting, both proteins eluted together from the gel-filtration column with an apparent mass according to MALS analysis of 255.9 kDa (Fig. 3A), which agrees almost with the theoretical calculated mass of NAGK-PII complex. However, with both variants, we were unable to detect any indications of a stable *Se*NAGK-PII complex, since *Se*NAGK eluted as free hexamer with an apparent mass of 193.8 kDa (Fig. 3A). In agreement, the corresponding fractions as analyzed by SDS-PAGE contained *Se*NAGK without any traces of PII (Fig. 3B). This experiment confirmed that the physical interaction between *Se*NAGK and different variants of PII protein is weak and the weak complexes dissociate during the gel-filtration.

Additionally, we assessed whether binding of *Se*PII (K58N) and (K58N/L56K) variants to *Se*NAGK could be measured by surface plasmon resonance (SPR) spectroscopy (Fig. 3C). The His-tagged *Se*NAGK was immobilized on a Ni-NTA sensor chip and *Se*PII variants (K58N) and (K58N/L56K), as well as *Se*PII (WT) protein, were injected sequentially. No response signal could be detected in binding assays between *Se*NAGK and the two PII variants (K58N) or (K58N/L56K), implying that the interaction between these *Se*PII variants and *Se*NAGK is too weak to be detected by SPR in comparison to *Se*PII (WT), which forms a strong complex with *Se*NAGK (Fig. 3C).

### K58N mutation influences negatively PII-PipX complex formation

**Figure 4:**
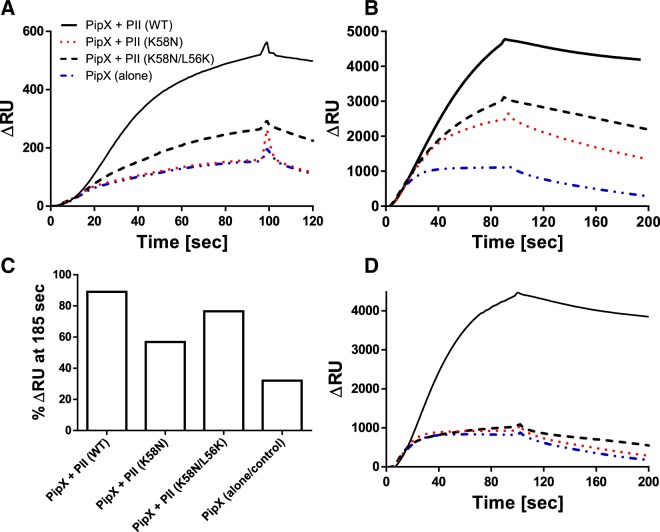
Interaction of PII variants (K58N) or (K58N/L56K) with PipX in presence or absence of effector molecules. His_6_-PipX (500 nM) was injected to the Ni-NTA loaded sensor chip in the absence of PII (blue dashed line) or in presence of either 500 nM PII (WT) (black line), or variants (K58N) (red dashed line) or (K58N/L56K) (black dashed line) (**A**) without effectors, (**B**) in presence of ADP (3 mM), and (**D**) in presence of ATP (1 mM). (**C**) Response signal in % at time *185* *s* (90 s after the end of the injection) of PipX injections in absence or presence of different PII variants and ADP at 3 mM (as part B). The response signal before the end of the injection (association phase) at *t: 95* is normalized to 100%. The maintained signal at *t: 185* *s* is an indicator for the stability of the complex.

Another major interacting partner of the PII signaling network in cyanobacteria is PipX^25^. In the absence of 2-OG, three PipX monomers can bind to one PII trimer^15^. The crystal structure of the PII-PipX complex revealed that the T-loop of PII acts as an antenna that attracts the PipX monomers^15,26^. Unlike the bent conformation of the T-loop in NAGK-PII complex, the T-loop in the PipX-PII complex is in an extended conformation^15^, resembling PII in its ADP-complex^7^. PII in the Mg^2+^-ATP-2-OG complex is unable to bind PipX^1,25^. Since the PII (K58N) mutation impaired ADP binding (Fig. 1), we assumed that the K58N variant may affect PII-PipX complex formation. We analyzed the formation of PII-PipX complexes by SPR spectroscopy, using an indirect assay, as described previously^8,16^. When PipX is incubated with PII prior the injection on the sensor chip, PII-PipX complex formation increases the binding of His_6_-PipX to the sensor chip due to mass increase and PipX trimerization (Fig. 4A). In absence of the effector molecules, the PII (K58N) variant was not able to increase PipX binding above the background level (binding of His_6_-PipX to the sensor in absence of PII), while the PII (K58N/L56K) was able to partially restore the binding of His_6_-PipX to the sensor chip, although not as efficient as PII (WT) (Fig. 4A). In presence of 3 mM ADP, the PII (K58N) variant regained the ability to at least partially interact with PipX (Fig. 4B). Again, the PII (K58N/L56K) variant showed a stronger interaction with PipX than the PII (K58N) variant but still weaker than PII (WT) (Fig. 4B). To compare the effect of PII variants on the dissociation rate of the complexes from the sensor chip (which is a good indicator of the efficiency of PII-PipX interaction), the dissociation curves were normalized to the RUs at the end of the association phase (taken as 100%) (Fig. 4C). In presence of ADP, the PipX-PII (K58N/L56K) variant complex dissociated slowly, in comparable way to the PipX-PII (WT) complex, while the PipX-PII (K58N) complex dissociated faster (Fig. 4C), confirming again that L56K could compensate the loss of Lys58. In presence of 1 mM ATP, PII (WT) bound to PipX weaker than in presence of ADP, in agreement with previous reports^8,16^, while both PII variants (K58N) and (K58N/L56K) were not able to efficiently interact with PipX (Fig. 4D).

### The T-loop of the PII (I86N) variant allows PipX interaction

**Figure 5:**
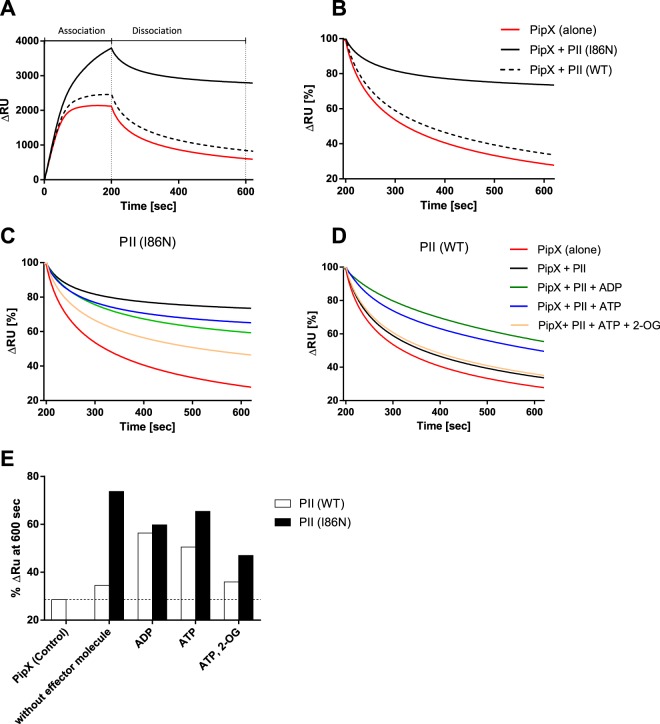
Indirect SPR analysis of PII-PipX complex formation in presence or absence of effector molecules. (**A**) His_6_-PipX (500 nM) was injected to a Ni-NTA loaded sensor chip in absence of PII (red line) or in presence of 100 nM PII (WT) (black dashed line) or 100 nM PII (I86N) (black line), without effectors. The injection phase of 200 s was followed by 400 s dissociation. (**B**) Dissociation from the sensor of His_6_-PipX (red line) alone or in presence of PII (WT) (black dashed line) or PII (I86N) (black line). The response signal at the end of the injection time *(200* *s)* was normalized to 100%. (**C,D**) Dissociation assay as described in (**B**) for PipX-PII (I86N) (**C**) or PipX-PII (WT) (**D**) complexes in presence of different combinations of effector molecules: Without effectors (black line), in presence of 1 mM ADP (green line), 1 mM ATP (blue line) or 1 mM ATP/ 1 mM 2-OG (orange line). PipX without PII in absence of effectors was used as control (red line). (**E**) Response signal in % at time *600* *s* (400 s after the end of the injection) of injections of PipX complexed with PII (WT) or PII (I86N) in presence or absence of effectors. The remaining signal after dissociation at time *600* *s* is an indicator for the stability of the complex.

Previously, we reported a NAGK hyper-activating variant PII (I86N)^6,22^. The I86N substitution causes the T-loop of PII to adopt a compact conformation through formation of hydrogen bond between the backbone oxygen of Thr43 and the amido group of Asn86 resulting in a contraction of the T-loop^6^. As consequence, the PII (I86N) variant binds constitutively to NAGK. However, the interaction of PII (I86N) variant with PipX has not been analyzed before. Here, we used the same indirect SPR assay to determine PII-PipX complex formation by determining binding of the complex to the Ni-NTA sensor surface. When the PII (I86N) variant or PII (WT) was pre-incubated with PipX in the absence of effector molecules, the PII (I86N) variant promoted a stronger binding of PipX to the Ni-NTA sensor chip surface and the complex dissociated slower than with PII (WT) (Fig. 5A). To quantitatively compare the effect of the two PII proteins on the dissociation of the complexes from the sensor chip, the dissociation curves were normalized to the RUs at the end of the association phase (taken as 100%) (Fig. 5B). The percent RUs remaining bound to the chip after 400 s of dissociation were then taken as a proxy for PII-PipX interaction and used to quantify the effect of different effector molecules (Fig. 5B–E).

Different effector molecules were tested on PII-PipX complex stability. As expected, a strong positive effect of ADP on the interaction of PII (WT) with PipX, was obtained^8^ (Fig. 5D,E). By contrast, binding of the PII (I86N) variant to PipX was negatively affected by ADP (Fig. 5C–E). ATP showed for the PII (WT) protein a slightly lower stability of the complex than in the ADP-complexed state whereas the PII (I86N) variant interacted stronger with PipX in the ATP state than with ADP (Fig. 5C–E). As expected, 2-OG in presence of ATP impaired PipX-PII (WT) complex formation^8,16^ (Fig. 5D). An inhibitory effect of ATP and 2-OG on the PII-PipX complex was also visible with the PII (I86N) variant, however not as strong as with PII (WT) protein (Fig. 5C–E). This agrees with the decreased affinity of the PII (I86N) variant towards 2-OG^6^. Taken together, these data demonstrated that the PII (I86N) variant is very efficient in complex formation with PipX and that this binding does not require positive stimulation by ADP as is the case with PII (WT). It appears that the T-loop of this variant is not permanently fixed in the bent conformation but is still flexible enough to adopt the extended conformation for PipX binding, highlighting the defining role of PII target proteins for the actual T-loop conformation.

## Discussion

Previous structural analysis of PII proteins in various complexed states showed that Lys58 plays an important role in the function of PII^5,7,23,24^. However, the Lys58 residue is not involved in direct interactions with ADP or ATP, but seems to be important to stabilize the position of the B-loop via a hydrogen bond to residue Gly87 (PDBs: 2XUL, 3MHY, and 2XBP). Binding of ADP induces conformational changes within the surface exposed T-loop enabling Lys58 to interact with Gln39 at the base of the T-loop^23^. Thereby, Lys58 anchors the T-loop in the ADP bound conformation via hydrogen bond to Gln39 (PDB: 4CNZ)^7,23^. Further, it anchors also the T-loop in the bent conformation of the PII-NAGK complex via a salt-bridge to Glu44 (PDBs: 2V5H and 2XBP)^6,12,23–25^.

Remarkably, binding of 2-OG is coordinated mainly by the highly conserved residues of PII proteins Lys58 and Gln39^5,24^. When 2-OG breaks the interaction between PII and NAGK, the Lys58-Glu44 interaction in PII is replaced by a new salt bridge interaction between Lys58 and 2-OG (PDBs: 2XUL and 3MHY). Elegantly, the C5 carboxyl group of 2-OG quite precisely replaces the Glu44 carboxyl group, such that Lys58 does not need to change its orientation. Therefore, we expected the K58N variant to be defective in both 2-OG binding and in anchoring the T-loop in the NAGK-bound conformation.

Complex formation of PII with NAGK follows a two-step mechanism^6^: first, an encounter complex involving the B-loop of PII is formed. This involves a contact between Arg233 of NAGK and Glu85 of PII. This interaction breaks a salt bridge between Glu85 and Arg47 in the T-loop of PII, which is now free and can adopt a bended structure. The T-loop bended confirmation is stabilized by a new salt bridge between Lys58 and T-loop residue Glu44. In the second step, the bended T-loop deeply inserts into the NAGK clefts to form the tight complex. Apparently, the mutation of Lys58 still allows bending of the T-loop and insertion into the NAGK clefts (with the help of the other hydrophobic interactions between Ile229, Ile253, and Ala257 of NAGK and Phe11 and Thr83 of PII)^12^, however, the overall stability of the complex is weakened and feedback inhibition by arginine is almost not affected (Fig. 2B). Therefore, we conclude that a tight complex between PII and NAGK is required to relief NAGK from arginine feedback inhibition, whereas the weak complex is sufficient to activate the NAGK in absence of arginine (Fig. 2A).

The *in vitro* assays confirmed that Lys58 is a key residue for proper signaling function of PII via affecting the sensory properties of ATP/ADP/2-OG as well as the interactions with NAGK and PipX. The PII (K58N) variant is still able to bind ATP but lost the ability to bind 2-OG and influence ADP binding negatively. Consistent with our data, previous results demonstrated that a PII (K58M) variant was not able to bind to 2-OG^5^. The Lys58 residue is not involved in direct interactions with adenyl nucleotides (ATP and ADP) but rather stabilizes the T- and B-loops via hydrogen bonds to Gln39 and Gly89, respectively. Therefore, we assume that the defect in anchoring the T- and B-loops in the PII (K58N) variant is most likely the cause for changing the affinities of ATP and ADP in comparison to *Se*PII (WT) (Table 1). The effect of the Lys58 mutation in the destabilization of PII T-loop is probably the dominating effect and explains, why the Lys58 mutation impairs the NAGK and PipX interactions, which are mediated by the T-loop of PII. Intriguingly, the addition of a compensatory mutation to K58N by replacement of the nearby Leu56 to Lys partially restores the ability of this PII variant to interact with NAGK and PipX. This compensatory effect indicates that the Leu56 to Lys mutation might restore a productive T-loop bent conformation, possibly by approaching the Glu44 residue of the T-loop (model from PDBs: 2XBP and 2V5H).

In cyanobacteria, the regulation of nitrogen metabolism mainly depends on interaction network composed of the signal-transduction protein PII, the transcription factor NtcA and its co-activator PipX (PII interacting protein X)^25^. The crystal structure of PII (I86N)^6^ (PDB: 2XBP) showed an almost identical backbone as PII (WT). However, the T-loop adopts a compact conformation, which is a structural mimic of PII in NAGK complex^6^. Hence, the PII (I86N) variant strongly activates NAGK *in vitro* and *in vivo*, causing huge accumulation of arginine and cyanophycin^6,22^. Therefore, we assumed that this variant was unable to interact with other PII interaction partners, which would influence the NtcA regulon through impaired PipX interaction. To our surprise, we detected strong interaction between the PII (I86N) variant and PipX. SPR spectroscopy confirmed that the PII (I86N) maintains the ability to bind PipX. In absence of effector molecules, the PII (I86N) variant showed even stronger interaction with PipX than PII (WT). Furthermore, the PII (I86N) variant did not require positive stimulation by ADP to form a stable complex as it is the case of PII (WT). It was previously shown that the PII (I86N) variant did not respond to 2-OG in complex with NAGK^6^. Nevertheless, an inhibitory effect of ATP and 2-OG on PII (I86N)-PipX complex was observed, although not as strong as in the case of the PII (WT) protein. Altogether, the interaction of PipX with PII (I86N) is different than with PII (WT), and complex stability is generally higher compared to PII (WT). Due to the stronger sequestration of PipX by PII (I86N), we speculate that PII (I86N) might tune down PipX mediated activation of NtcA *in vivo*. This could explain the delayed nitrogen starvation response observed in the *Synechocystis* strain BW86 harboring the PII (I86N) variant^22^. As a future perspective, a transcriptomic analysis would be informative to characterize the *in vivo* influence of PII (I86N) on the NtcA regulon, in response to nitrogen limitation conditions.

Altogether, our study provides new insights into the signaling function of PII proteins and sheds some lights on the plasticity of the PII core-structure. This is, for example, demonstrated by the partial compensation of the Lys58 mutation, by the Leu56 to Lys mutation indicating the plasticity of the PII body. This flexibility of PII body could be used in protein engineering to design new proteins or sensors with new desired characters. Notably, the PII variant (I86N) is able to bind citrate as a new effector molecule^27^, and was used to engineer a cyanobacterial strain that produces huge amounts of the biopolymer cyanophycin^22^. In other studies, the trimeric architecture of PII proteins was used to engineer 2-OG^28,29^ or ATP^30^ sensors. Since the PII variants (K58N) and (K58N/L56K) no more bind 2-OG but still respond to ATP and ADP, it would be interesting to characterize their *in vivo* impact on cyanobacterial physiology with respect to the known PII targets (NAGK^12^, PipX^15^, ACCase^14^, N-transporters^19^). In a future prospective, such variants may be useful for re-directing cyanobacterial metabolism for biotechnological applications.

## Material and Methods

### Cloning, overexpression and purification of recombinant PII variants, NAGKs, and PipX proteins

Recombinant Strep-tagged PII proteins (WT and I86N variant) from *S. elongatus* were prepared as described previously^6,10,11,13^. The strep-tagged PII variants (K58N) and (K58N/L56K) were generated by amplifying the whole PII(WT)-pASK-IBA3 plasmid using site-directed mutagenesis primers. The PII(K58N)-pASK-IBA3 plasmid was generated using 1848_Fw: GGTTGAGTTTTTGCAAAATCTGAAGCTCGAG and 1849_Rv: GTGTATTCCGAGCCGCGATAGC primers, while the PII(K58N/L56K)-pASK-IBA3 plasmid was generated using 1850_Fw: GGTTGAGTTTAAGCAAAATCTGAAGCTCG and 1849_Rv primers. The generated plasmids were verified by sequencing. The pASK-IBA3 based plasmids were transformed and overexpressed in PII deficient *E. coli* strain RB9060^9^. The recombinant strep-tagged PII proteins were purified using affinity chromatography as described previously^3,6,9–11^. The His_6_-tagged *Se*NAGK, *Sc*NAGK and PipX were overexpressed in *E. coli* BL21(DE3) and purified via affinity chromatography using His-Trap column (GE Healthcare) as described previously^9–11,16^.

### Isothermal titration calorimetry (ITC)

ITC experiments were performed using VP-ITC microcalorimeter (MicroCal) in a buffer composed of 50 mM Tris-HCl (pH 8.0) and 200 mM NaCl at 20 °C, according to previous publication^9^. The row calorimetric data were fitted according to three-sequential binding sites models using MicroCal Origin software^3,9^.

### Coupled NAGK activity assay

To assess the influence of PII variants (K58N and K58N/L56K) on the NAGK activity, we used our standard NAGK-coupled assay in which the formation of ADP is coupled to the oxidation of NADH using the auxiliary enzymes pyruvate kinase and lactate dehydrogenase as described previously^9,10,13^. The specific activity of NAGK was expressed in µmol/min/mg.

### Surface plasmon resonance (SPR) spectroscopy for determination of PII-NAGK and PII-PipX complex formation

The SPR experiments were performed using a BIAcore X biosensor system (GE Healthcare) as described previously^6,8,10,13^ at 25 °C with HBS-Buffer (10 mM Hepes pH 7.5, 150 mM NaCl, 1 mM MgCl_2_ and 0.005% (w/v) Nonidet P40). For a typical assay, 100–1000 nM Strep-tagged PII (WT or variants) proteins were pre-incubated in the absence or in presence of effectors (1 or 3 mM ADP; 1 mM ATP; 1 mM ATP/2-OG) for 10 min at room temperature. To assess the ability of PII variants (K58N, K58N/L56K, and I86N) to bind to PipX, an indirect assay was used as described previously^8,16^. Briefly, the assay measures the interaction of His_6_-PipX with a Ni-NTA sensor chip in the presence or absence of PII. Upon PII interaction, monomeric PipX protein trimerizes at the PII interaction surface, which leads to a stabilization of the His_6_-PipX interaction with the Ni-NTA surface. Furthermore, the additional mass of the PII protein leads to an increased SPR signal. For a binding assay, 500 nM His_6_-PipX was added to the pre-incubated Strep-tagged PII proteins and further incubated for 10 min at room temperature. The PipX-PII mixture was injected to both of flow cell 1 (FC1; without Ni^2+^) and flow cell 2 (FC2; Ni^2+^-loaded) and the resonance difference between specific binding (to FC2) to unspecific binding (to FC1) was recorded. FC1 was used as a background control. As control, His_6_-PipX was injected to the chip in absence of PII. The Injection (association) phase was at a flow rate of 15 µl/s; followed by dissociation at a flow rate of 15 µl/s. ΔRUs at the end of injection phase (as indicated), were normalized to 100% to compare the different dissociation rates (i.e. the difference in RU in % at the end of dissociation phase as compared to 100% RU at the beginning of dissociation). The resulting % ΔRUs after 400 s or 90 s of dissociation are an indicator for complex stability. To discriminate the PII variants (K58N and K58N/L56K) from PII (WT), we used high ADP concentrations (3 mM) to ensure saturation of PipX-PII complex^8^, however it’s not of physiological relevance.

To evaluate the interaction of PII variants (K58N and K58N/L56K) with His_6_-NAGK, standard SPR experiments were performed as described previously^6,10,13^. Briefly, His_6_-NAGK was immobilized on a Ni^2+^-loaded NTA chip to flow cell 2 (FC2), then PII protein was injected on FC1 and FC2 and the difference in RUs between FC2 and FC1 (ΔRU) was recorded, which represents the specific interaction between PII and NAGK. To load fresh proteins on to the NTA sensor chip, bound proteins were removed by injecting 25 μl of 0.4 M EDTA pH 7.5 (flow rate 15 µl/s). Subsequently, 10 µl of 5 mM Ni_2_SO_4_ (flow rate 10 µl/s) was injected to FC2.

### Size exclusion chromatography coupled to multiangle angles light scattering (SEC-MALS) analysis

SEC-MALS was carried out using an Äkta chromatography system (GE Healthcare) connected to a MALS detection system at room temperature. SEC-MALS experiments were done as described previously^13,31^ on a 24 ml Superose 6 Increase 10/300 GL column (GE Healthcare) to which an Optilab T-rEX refractometer and a miniDawn Treos detector (Wyatt Technology Corp.) were attached. The runs were performed at a flow rate of 0.5 ml/min after equilibration of the column with running buffer (100 mM Tris-HCl/pH 7.8, 300 mM NaCl, 2 mM MgCl_2_, 2% glycerol). The PII proteins were mixed with NAGK (molar ratio of 3:1 PII trimers to NAGK hexamers) for 10 min at room temperature, then 100 µl of the mixture was applied to the SEC column. The elution volume was plotted against the UV signal and against the molecular mass derived from the light scattering data. Data analysis and molecular weight calculations were done using ASTRA software (Wyatt). The elution fractions were collected and subjected to SDS-PAGE for analysis.

## Supplementary information

Supplementary Information
