# Supplementary material for: Tuning the in vitro sensing and signaling properties of cyanobacterial PII protein by mutation of key residues
Source: Sci Rep. 2019 Dec 12;9:18985. doi: 10.1038/s41598-019-55495-y (PMC6908673; doi:10.1038/s41598-019-55495-y)
Supplement: Supplementary file 1 — Supplementary Information [file 41598_2019_55495_MOESM1_ESM.pdf]

Supplementary information for:

Tuning the *in vitro* sensing and signaling properties of cyanobacterial PII protein by mutation of key residues

Authors: Khaled A. Selim[a,1], Michael Haffner[a], Björn Watzer[a], Karl Forchhammer[a]

Affiliations:

[a] Interfaculty Institute of Microbiology and Infection Medicine, Department of Organismic Interactions, University Tübingen, Auf der Morgenstelle 28, 72076 Tübingen, Germany.

Supplementary Figures

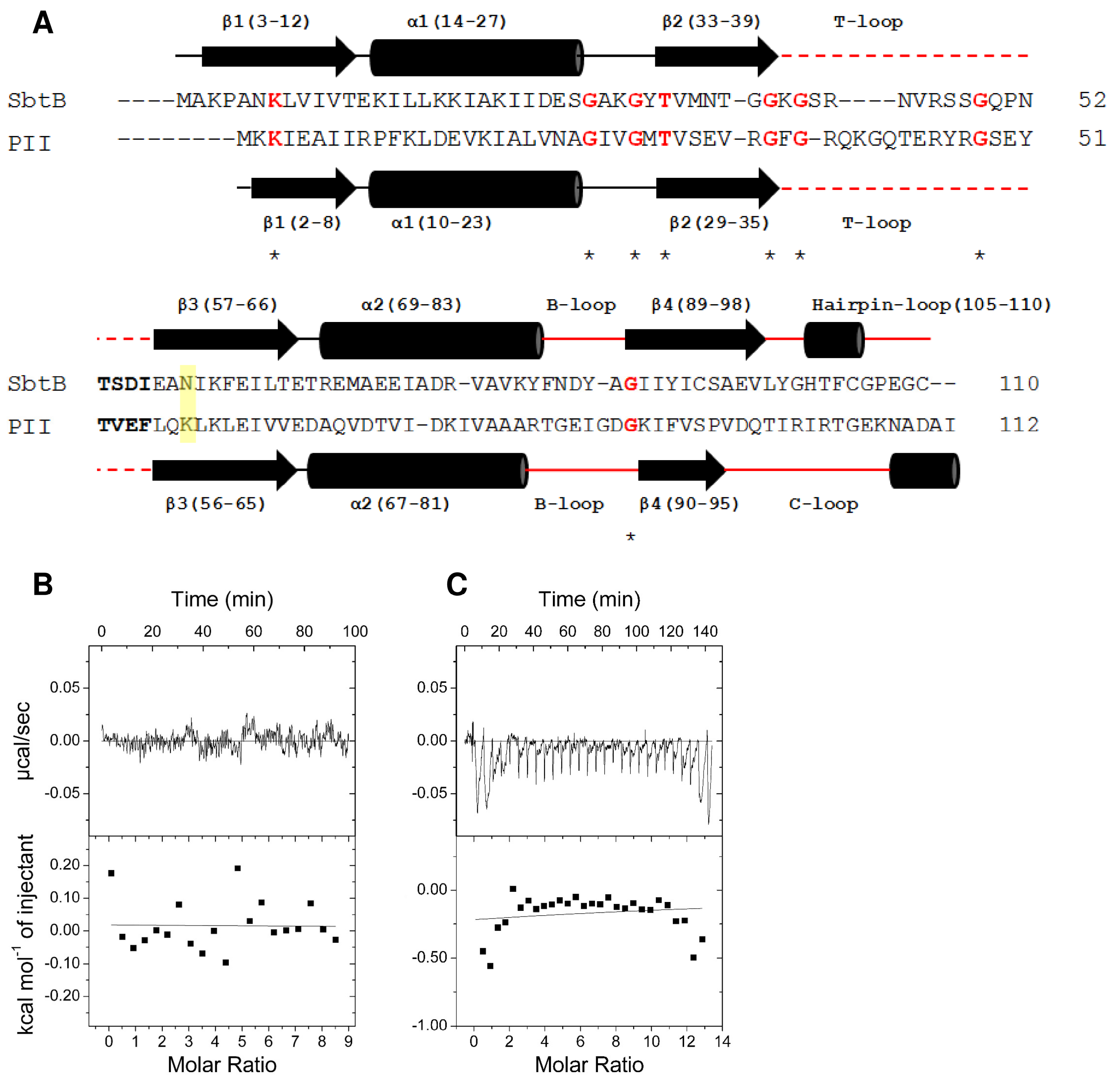

Supplementary Fig. S1. (A) Structure-based sequence alignment of *Se*PII protein (PDB: 2XUL) and PII-like protein SbtB (PDB: 5O3R), shows replacement of Lys58 in SePII by Asn in SbtB (highlighted in yellow). Secondary structures of SbtB and *Se*PII proteins are shown above and below the alignment, respectively. T-loops are indicated with dashed lines; highly conserved residues are indicated by asterisks. **(B & C)** ITC analysis for binding of 2-OG (2 mM) to 33 µM of PII variants: **(B)** PII (K58N) and **(C)** PII (K58N/L56K) variants in presence of 1 mM Mg^{2+}-ATP. The upper panels show the raw isothermal data in the form of the heat effect during the titration of PIIs solution with ligands.

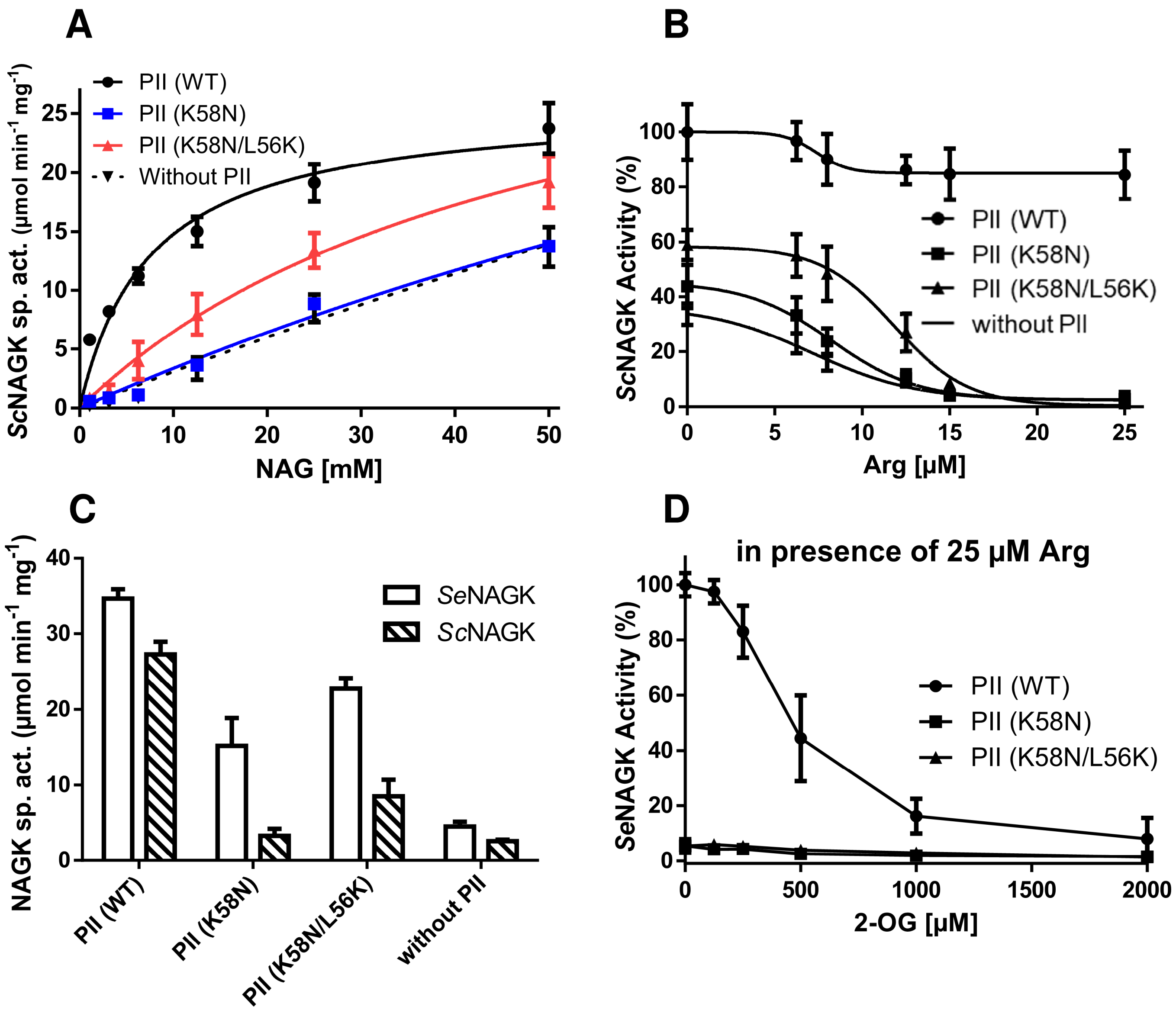

Supplementary Fig. S2. Response of NAGK activity towards *Se*PII (WT) or variants (K58N) and (K56N/L56K). (A) The catalytic activity of *Sc*NAGK with or without different PII variants, as indicated. NAG was used as a variable substrate. **(B)** Arginine-feedback inhibition of *Sc*NAGK activity in the presence or absences of different variants of PII protein, as indicated. Data were fitted according to a sigmoidal dose-response curve using a GraphPad Prism to estimate the IC_{50} for arginine. **(C)** The catalytic activity of different NAGKs with or without different variants of PII protein in presence of 12.5 µM of arginine, as indicated. **(D)** Effect of 2-OG on PII-promoted activation of *Se*NAGK in presence of 25 µM arginine, as indicated. SD as indicated by error bars, represents triplicate independent measurements.
